# Anticancer Activity of Au/CNT Nanocomposite Fabricated by Nanosecond Pulsed Laser Ablation Method on Colon and Cervical Cancer

**DOI:** 10.3390/mi14071455

**Published:** 2023-07-20

**Authors:** Abbad Al Baroot, Khaled A. Elsayed, Firdos Alam Khan, Shamsuddeen A. Haladu, Filiz Ercan, Emre Çevik, Q. A. Drmosh, M. A. Almessiere

**Affiliations:** 1 Department of Basic Engineering Sciences, College of Engineering, Imam Abdulrahman Bin Faisal University, Dammam 31441, Saudi Arabia; 2 Department of Stem Cell Research, Institute for Research and Medical Consultations (IRMC), Imam Abdulrahman Bin Faisal University, Dammam 31441, Saudi Arabia; 3 Department of Physics, College of Science, Imam Abdulrahman Bin Faisal University, Dammam 31441, Saudi Arabia; fsercan@iau.edu.sa (F.E.);; 4 Basic & Applied Scientific Research Centre, Imam Abdulrahman Bin Faisal University, Dammam 31441, Saudi Arabia; 5 Department of Biophysics, Institute for Research and Medical Consultations (IRMC), Imam Abdulrahman Bin Faisal University, Dammam 31441, Saudi Arabia; ecevik@iau.edu.sa; 6 Department of Materials Science and Engineering, King Fahd University of Petroleum & Minerals, Dhahran 31261, Saudi Arabia; 7 Interdisciplinary Research Centre for Hydrogen and Energy Storage (HES), King Fahd University of Petroleum and Minerals (KFUPM), Dhahran 31261, Saudi Arabia

**Keywords:** gold nanoparticles, MWCNT, laser ablation, colon cancer, cervical cancer

## Abstract

Gold nanoparticles (AuNPs) and carbon nanotubes (CNTs) are increasingly being investigated for cancer management due to their physicochemical properties, low toxicity, and biocompatibility. This study used an eco-friendly technique (laser synthesis) to fabricate AuNP and Au/CNT nanocomposites. AuNPs, Au/CNTs, and CNTs were tested as potential cancer nanotherapeutics on colorectal carcinoma cells (HCT-116) and cervical cancer cells (HeLa) using a 3-(4,5-dimethylthiazol-2-yl)-2,5-diphenyl-2H-tetrazolium bromide (MTT) assay. In addition, the non-cancer embryonic kidney cells HEK-293 were taken as a control in the study. The cell viability assay demonstrated a significant reduction in cancer cell population post 48 h treatments of AuNPs, and Au/CNTs. The average cell viabilities of AuNPs, Au/CNTs, and CNTs for HCT-116 cells were 50.62%, 65.88%, 93.55%, and for HeLa cells, the cell viabilities were 50.88%, 66.51%, 91.73%. The cell viabilities for HEK-293 were 50.44%, 65.80%, 93.20%. Both AuNPs and Au/CNTs showed higher cell toxicity and cell death compared with CNT nanomaterials. The treatment of AuNPs and Au/CNTs showed strong inhibitory action on HCT-116 and HeLa cells. However, the treatment of CNTs did not significantly decrease HCT-116 and HeLa cells, and there was only a minor decrease. The treatment of AuNPs, and Au/CNTs, on normal HEK-293 cells also showed a significant decrease in cell viability, but the treatment of CNTs did not produce a significant decrease in the HEK-293 cells. This study shows that a simplified synthesis technique like laser synthesis for the preparation of high-purity nanomaterials has good efficacy for possible future cancer therapy with minimal toxicity.

## 1. Introduction

Nanomaterials are increasingly explored for use in biomedical applications owing to their unique and outstanding properties, which render them suitable for developing new and enhanced products for diagnosis and therapy. Along this line, nanotechnology has been employed to remedy various metabolic and pathological disorders, including inflammation, cancers, arthritis, liver, HIV, etc. [1]. Nanomaterial-mediated cancer therapy is advantageous over other conventional therapeutic modalities as it averts their limitations, such as lack of specificity of the used chemotherapeutics, in the sense that they damage other normal tissues, the occurrence of chemotherapy drug resistance, etc. 

Colorectal cancer (CRC) ranks as the third most occurring cancer globally, associated with morbidity and mortality and is reported to be a major cause of death and morbidity in Western countries as its occurrence is higher in developed countries [2,3]. Depending on its stage, CRC is commonly treated by surgical resection and/or adjuvant chemotherapy [4]. Likewise, cervical cancer is a devastating disease linked to high mortality (fourth most in females), with an estimated 570,000 diagnosed women in 2018 and about 311,000 deaths [5]; it is caused by infection with human papillomaviruses [6]. Standard treatments for cervical cancer involve chemoradiotherapy, surgery, immunotherapy, and targeted therapy [7]. Despite these traditional cancer therapies, the survival rate of patients is still below expectations [2,6]. Hence, there is a need to devise robust treatment strategies. Consequently, nanotechnology offers suitable intervention options, ranging from tumor screening to the fabrication of targeted drug delivery materials and advanced treatment modalities [2,8]. 

Gold nanoparticles (AuNPs) are among the most extensively investigated materials with promising performance in cancer diagnosis and therapy [9]. They are small in size, nontoxic, biocompatible, and function as anticancer drug delivery vehicles and in the visualization of tumors [10,11,12,13]. AuNPs can be used in light-based clinical (photodynamic) therapies since they can absorb visible light and efficiently deliver wavelength-specific energies to the target [14]. A recent review discussed the role of AuNP therapeutic platforms as a clinically safer and more effective cancer treatment [15]. Carbon nanotubes (CNTs) are also widely investigated for applications in nanomedicine because of their fascinating characteristics while possessing biocompatibility and low toxicity [16,17]. CNTs mainly act as drug carriers in cancer therapy. For example, in an in vivo study on mice, the cancer chemotherapy drug paclitaxel was conjugated to functionalized CNTs resulting in improved efficacy in mitigating tumor growth [17]. Furthermore, in an attempt to overcome multidrug resistance to cancer chemotherapy medications, antibody-functionalized CNTs were loaded with doxorubicin cancer medication, where there exists observed recognition of multidrug-resistant human leukemia cells, while enhancing the loading and controllable release of the drug [18]. Another CNT-derived drug delivery platform was developed by attaching the antitumor drug 10-hydroxy camptothecin to the CNTs using an ester linkage; the conjugate demonstrated superior antitumor performance both in vitro and in vivo [19].

Nanomaterials synthesis is commonly achieved by chemical, physical, and biological methods, with chemical techniques being the most widespread and conventional [20,21]. There is a growing demand to invent environmentally benign synthetic approaches that exclude toxic chemicals and solvents. To achieve that, recently, plant extracts have found increased usage in the green synthesis of nanomaterials, where they act as reducing and stabilizing agents. As for the green biomimetic approach, microorganisms are used, such as bacteria, fungi, and yeast, among others [11,22]. However, using microorganisms is cumbersome due to the need to maintain cell cultures [11]. Another efficient and environmentally friendly nanomaterial fabrication protocol is laser synthesis, where a solid target is ablated with a laser in a confined liquid medium such as deionized water [23,24,25]. In recent decades, this synthetic method has been increasingly pursued to fabricate nanocrystals like carbon-based materials, diamonds, immiscible alloys, and so on [26]. Laser synthesis demonstrates various advantages: chemically clean and simple without toxic by-products, feasible under ambient conditions of temperature and pressure, fast, etc., thus, enabling the selection of a suitable combination of liquid medium and solid targets to prepare compound nanomaterials with the desired applications [26,27,28,29]. Because laser synthesis affords high-purity nanostructures, which could fulfill the requirements of medical deployment, in this work, we report the laser synthesis of AuNP and Au/CNT nanocomposites in water. The fabricated materials demonstrate the merits of being prepared by a green technique and containing AuNPs as drug delivery probes, due to their small size, hydrophilic character, and other unique properties as well as incorporation of the promising nanocarrier CNTs due to both their ability to cross the plasma membrane and their high surface area. The impact of the prepared nanostructures was tested on two cancer cell lines, human colorectal carcinoma (HCT-116) and human cervical cancer cells (HeLa), on their viability and proliferation. Also, the non-cancer and healthy cell line (embryonic kidney cells, HEK-293) was taken as the control in the study.

## 2. Experimental Section

### 2.1. Materials 

This work used a gold metal target (Au) with high purity to fabricate AuNPs. Multi-wall carbon nanotubes (MWCNTs) were sourced from Cheap Tubes (>95% purity and electrical conductivity >100 S/cm).

### 2.2. Synthesis of Au/CNT Nanocomposite

**Figure 1:**
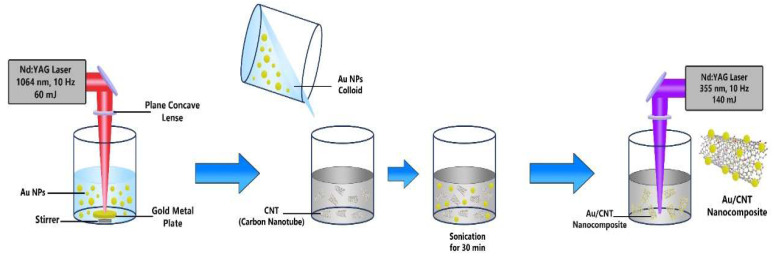
The synthesis process of the Au/CNT nanocomposites is demonstrated in this schematic diagram.

A pulsed laser ablation (PLA) technique was used to synthesize AuNP and Au/CNT nanocomposites. This experiment used two types of Q-switched Nd: YAG pulsed lasers model (PS- 2225, LOTIS TII Ltd., Minsk, Belarus) operation. The first type was set up with a wavelength of 1064 nm, 50 mJ pulse energy, and a 10 Hz repetition rate, while a pulse width of 10 ns, was used to prepare gold nanoparticles (AuNPs). The Au target was set at the bottom of a 10 mL glass vial filled with 4 mL of deionized water. and ablated for 15 min as investigated earlier in Ref. [30]. The height of the water above the Au target was 12 mm. The obtained Au NPs as a product were kept in a clean vial to use later. The second type of laser was set up with a wavelength of 355 nm, 140 mJ pulse energy, and 10 Hz repetition rate, while a pulse width of 10 ns was used as another laser source to fabricate Au/CNTs after mixing the composite. An amount of 10 mL of deionized water containing 0.7 mg of AuNPs, with 20 mg of CNTs, was ablated by the second type of laser for 30 min in an 18 mL cylindrical glass vial to create the new product, Au/CNT nanocomposite. The focus of the UV laser beam was adjusted below the surface of the liquid to prevent high fluence that might have ablated the surface air interface and in turn avoided splashing of the liquid. Vigorous magnetic stirring was undertaken with the samples which were irradiated for 30 min. Figure 1 shows the work schematic of the pulsed laser ablation process. The prepared nanoparticles and the nanocomposite details were reported in our recent work [23,31,32,33,34].

## 3. Characterization of Nanocomposites

### 3.1. Morphological Analysis

UV–vis spectrophotometry was used to study the absorbance spectra of the Au/CNT nanocomposites with the SolidSpec-3700 apparatus at scanning wavelengths from 200 to 800 nm. 

Surface morphological analysis of nanostructures was investigated by a high-resolution transmission electron microscope (HRTEM) (Morgani 268) at 200 kV. Elemental distribution analysis of the samples was conducted using energy dispersive X-ray (EDS) spectroscopy (EDAX, Octane Elect EDS System).

The powder XRD patterns were obtained to study the crystal structures of CNT, Au, and Au/CNT samples by using a Rigaku Ultima IV powder diffractometer with CuK_α_ radiation (λ = 1.5406 Å) and a scan speed of 0.02°/min, in the 2θ range from 10° to 100° at room temperature. The crystalline phases were identified by comparing the diffraction patterns with those of the standard powder XRD files (JCPDS: Joint Committee on Powder Diffraction Standards and COD: Crystallography Open Database). 

### 3.2. Anti-Cancer Activity

Human colorectal carcinoma (HCT-116) and human cervical cancer cells (HeLa) were purchased from ATCC, USA, and were used to examine the impact of AuNP, Au/CNT, and CNT cancer cell viability [35,36]. In addition, healthy cell embryonic kidney cells, HEK-293, were also included in the study as the control. First, cells were seeded in the 96 well plates containing special media of Dulbecco’s Modified Eagle Medium (DMEM) and kept in a CO_2_ incubator. AuNPs, Au/CNTs, and CNTs (2.0 µg to 40 µg/mL) were added in each, containing HCT-116, HeLa, and HEK-293 for 48 h and processed for 3-(4,5-dimethylthiazol-2-yl)-2,5-diphenyl-2H-tetrazolium bromide (MTT) assay [37,38]. The AuNPs, Au/CNTs, and CNTs were not added in the 2 control wells. Thereafter cells were exposed to MTT (5.0 mg/mL) for 4 h, and the media were replaced with dimethyl sulfoxide (DMSO). The plates were examined under a plate reader supplied by Bio-Tek Instruments, USA, at 570 nm wavelength, and the optical density (OD) was obtained to calculate the percentage of cell viability. The data, presented in the graph as mean ± standard deviation obtained from triplicates, were statistically evaluated by GraphPad Version 6.0 Prism Software USA.

### 3.3. Apoptotic DAPI Staining

To examine the impact of the treatment of AuNPs, Au/CNTs, and CNTs on the nucleus of cancer cells, we stained cells with 4′,6-diamidino-2-phenylindole (DAPI) staining post-48 h with 40 µg/mL. The blue, fluorescent staining was examined under a confocal scanning microscope (Zeiss, Munich, Germany). In brief, cells were treated with paraformaldehyde and labeled with DAPI dye. 

## 4. Results and Discussion

**Figure 2:**
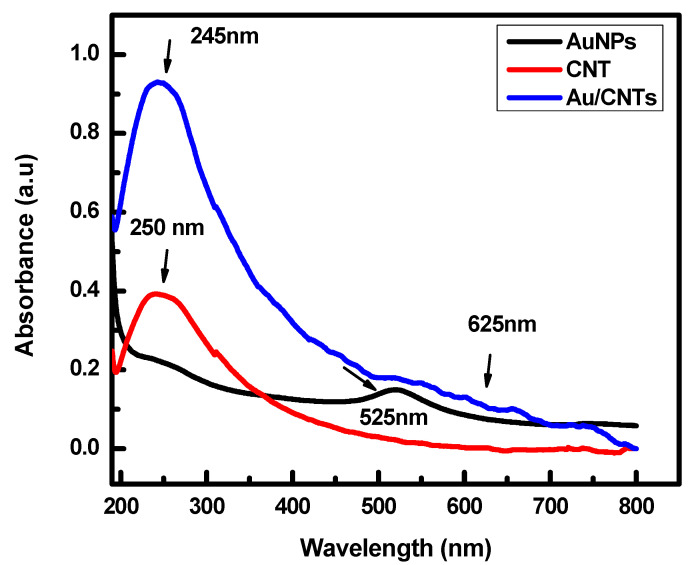
UV-vis absorption spectra of AuNP, CNT, and Au/CNT nanocomposites.

In this study, the absorption spectra of three samples are shown in Figure 2. These are the AuNP, CNT, and Au/CNT nanocomposites in the 200 to 800 nm range.

AuNPs demonstrate one major peak at 525 nm due to surface plasmon resonance (SPR), as reported by Chinh et al. [39]. The second sample, CNTs, shows one prominent peak at 250 nm, as investigated in the literature [40,41]. However, when AuNPs decorated the CNTs, we observed covalent bonds attributed to a sensible broadening and red shift of the Plasmon resonance band in the nanocomposite Au/CNTs as reported by Chinh et al. [39]. However, Au/CNTs showed a slight shift to 625 nm, which occurs due to aggregated bonds between two materials [39].

**Figure 3:**
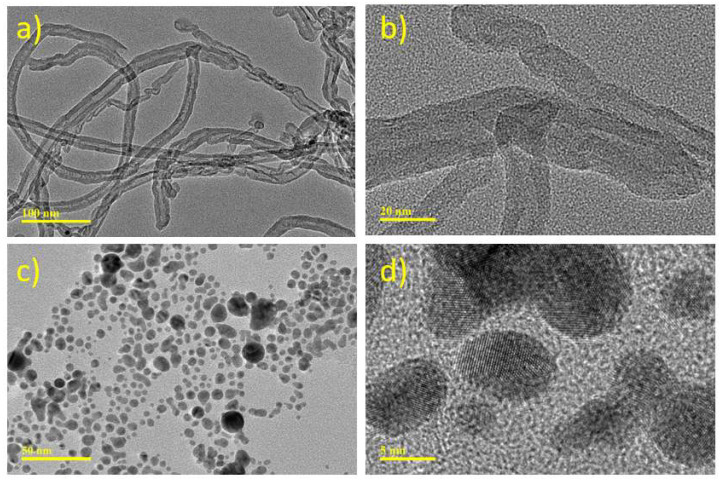
HRTEM Analysis of (**a**,**b**) CNT and (**c**,**d**) Au nanoparticles at different magnifications.

Figure 3a,b shows HRTEM images of CNT samples obtained at different magnifications. Tubular structures with an average diameter of 10 nm are visible in a uniform distribution in Figure 3a. The TEM image obtained at high magnification in Figure 3b confirms the tubular structure. TEM analysis of Au nanoparticles was achieved in different magnifications, as shown in Figure 3c,d. The images obtained show that the average particle size is between 3–20 nm and has a uniform distribution.

**Figure 4:**
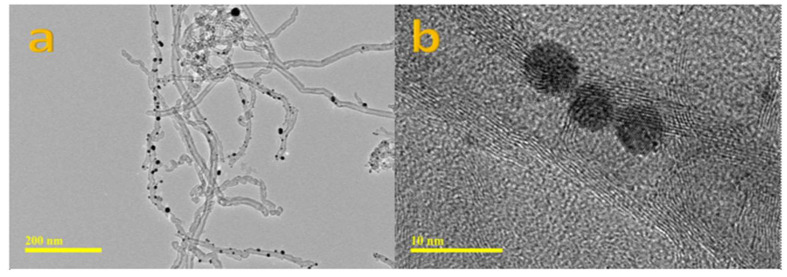
TEM Analysis of (**a**,**b**) Au nanoparticle decorated CNTs at different magnifications.

Detailed HRTEM analysis of AuNP-loaded CNT structures is shown in Figure 4a,b. As seen in Figure 4a,b, Au nanoparticles of different sizes are attached along the tubular structure. The attachment between CNT and Au nanostructures reveals the compatibility of both nanostructures. These features were also confirmed by HRTEM images obtained at high magnification, as shown in Figure 4b.

**Figure 5:**
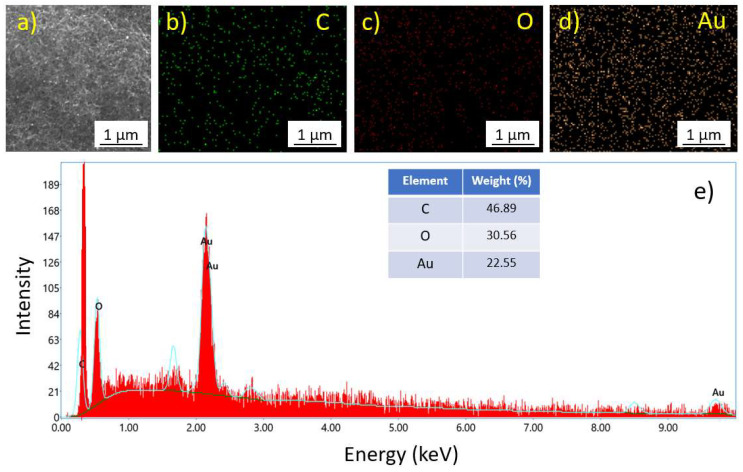
(**a**) SEM image of Au/CNT, EDS mapping of different elements, (**b**) carbon (C), (**c**) oxygen (O), and (**d**) gold (Au), (**e**) EDS spectrum of Au/CNT sample.

Elemental distribution of the Au/CNT sample was performed using energy-dispersive X-ray spectroscopy to examine the percentages of all respective elements in the sample. The SEM image was used to scan the EDX analysis to obtain the elemental distribution. Figure 5b–d shows the elemental mapping of C, O, and Au elements. The mapping confirms a uniform elemental distribution, and the elemental composition shows that 22% of Au doping was successfully achieved. 

**Figure 6:**
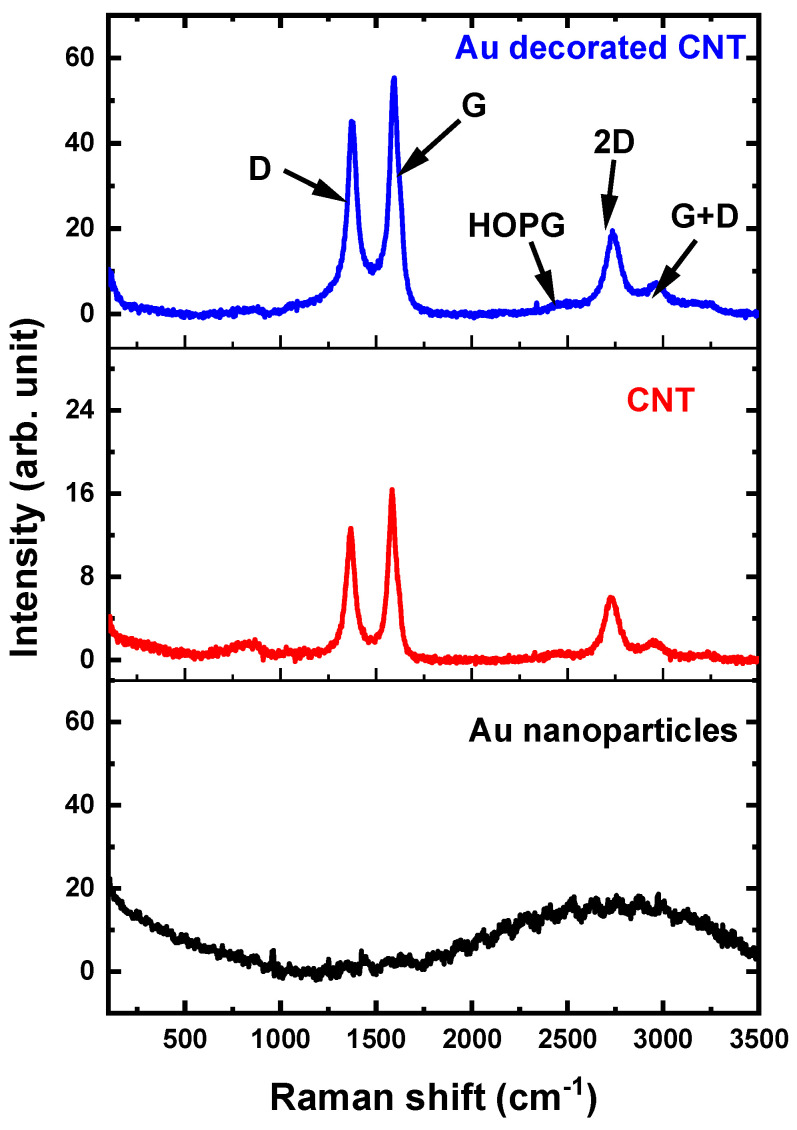
Raman spectra of Au nanoparticles, CNTs, and Au nanoparticle decorated CNTs prepared by pulsed laser ablation technique.

Figure 6 displays the Raman spectra of Au nanoparticles, CNTs, and Au nanoparticle decorated CNTs obtained using a LabRam HR evolution Raman Spectrometer Horiba Scientific at room temperature with a 455 nm laser light. As can be observed, at this excitation wavelength (455 nm), Raman scattering from pure Au nanoparticles was not observed. The Raman spectrum of CNTs demonstrates a typical disorder-induced D band (defect) at 1372 cm^−1^ and a G band (graphite band) at 1581 cm^−1^ indicating the formation of multi-walled CNTs [42]. Three other band peaks were observed at 2475 cm^−1^, 2728 cm^−1^, and 2959 cm^−1^ and attributed to highly oriented pyrolytic graphite (HOPG), 2D-band, and G + D band, respectively. The Raman spectrum of the Au nanoparticle decorated CNT sample shows that the D-band and G-band shifted to higher wavenumbers (1382, and 1591 cm^−1^, respectively). The shift in D-band and G-band is often ascribed to structural defects after functionalization, and a substantial charge transfer interaction between the AuNPs and CNTs, respectively [43,44]. In addition, one can observe that the intensity of the Raman signal of the Au nanoparticle decorated CNTs is higher than that of pure CNT, which is most likely due to the localized surface plasmon resonance effect caused by Au nanoparticles [45]. 

**Figure 7:**
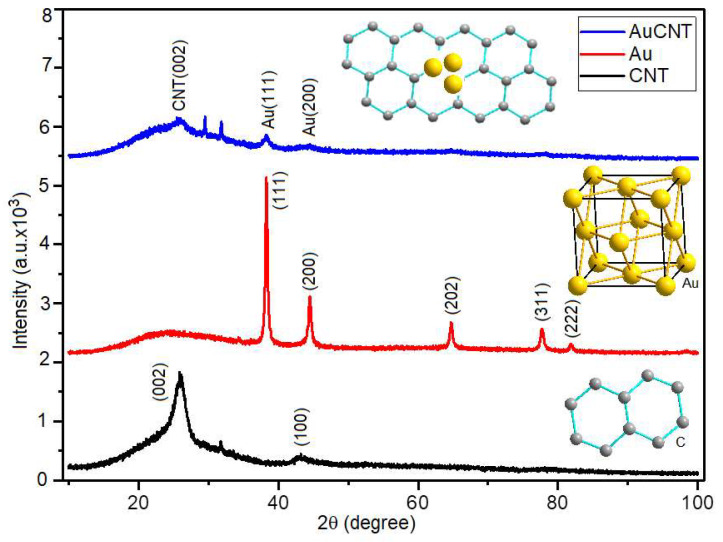
The powder XRD patterns of CNT, AuNP, Au/CNT nanocomposites.

CNT, Au and Au/CNT nano composite powder XRD patterns are shown in Figure 7. 

Carbon nanotubes (CNTs) are crystallized in the hexagonal crystal system based on COD: 96-120-0018, JCPDS No. 00-04-1487, and JCPDS No. 75-1621 [46,47,48]. The two characteristic diffraction peaks of CNT at 25.29° and 43.41° Bragg reflection angles are the diffractions of (002) and (100) lattice planes, respectively.

AuNP has a cubic crystal system and Fm-3m (No:225) space group (COD: 96-901-1614, JCPDS No. 04-0784) [49]. For Au, the hkl Miller indices corresponding to the 2θ values of 38.24°, 44.45°, 64.68°, 77.70°, and 81.86° were obtained as (111), (200), (202), (311), and (222), respectively. 

The XRD diffraction pattern of Au/CNT nanocomposite displays a broad diffraction peak from 2θ = 18.0°–28.0° of the nanocomposite corresponding to the (002) plane of CNT [50].

The characteristic diffraction reflections of crystalline AuNPs are evident in the Au/CNT combination [51]. In addition, in the Au/CNT composite, two reflections are observed between 28.0°–32.0° degrees. It can be seen from Figure 7 that the peak around 32.08° degrees initially accompanies the CNT diffraction peaks. Based on the XRD qualitative analysis results and literature review, it is thought that these two peaks may be of graphite–graphene oxide origin (COD: 96-100-0066) [52]. This behavior is supported by the literature on CNT-doped nanocomposites [37,53].

## 5. Impact of AuNPs, CNTs, and Au/CNTs on Cell Viability

**Figure 8:**
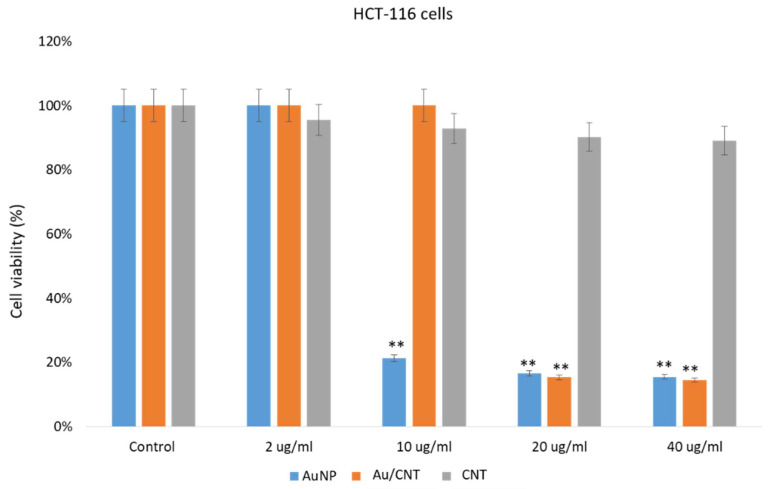
Colon cancer cell viability using MTT Assay: It shows the impact of AuNP, Au/CNT, and CNT treatment on HCT-116 cell viability post 48-h treatment. ** *p* < 0.001.

**Figure 9:**
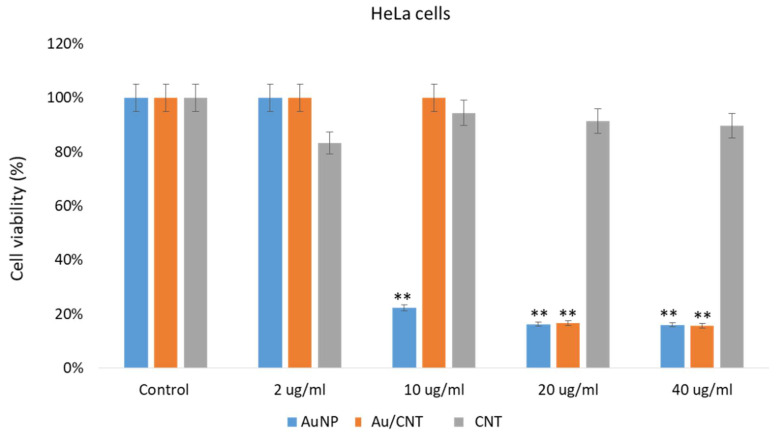
Cervical cancer cell viability using MTT Assay: It shows the impact of treatment of AuNPs, Au/CNTs, and CNTs on HeLa cell viability post 48 h treatment. ** *p* < 0.001.

The impact of AuNPs, CNTs, and Au/CNTs on HCT-116 and HeLa cells was examined. Post 48 h of treatment; we found a significant decrease in cancer cell post-treatments of AuNP and Au/CNT (Figure 8 and Figure 9). The treatment of AuNPs, and Au/CNTs showed strong inhibitory action on HCT-116 and HeLa cells (Figure 8 and Figure 9). However, the treatment of CNTs did not produce a significant decrease in the HCT-116 and HeLa cells; there was only a minor reduction (Figure 8 and Figure 9).

**Figure 10:**
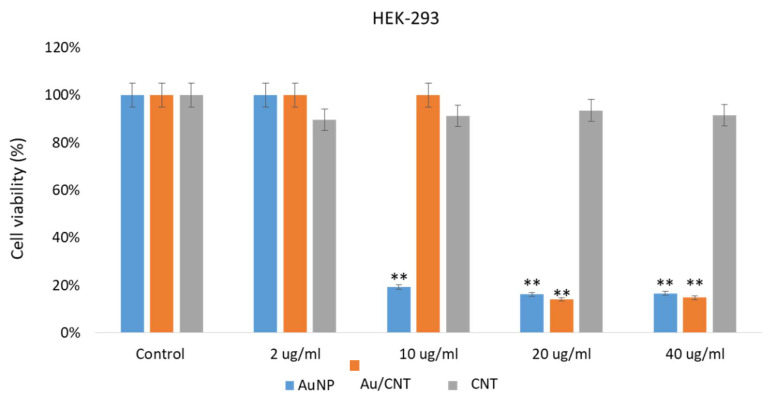
Non-cancerous cell viability using MTT assay: It shows the impact of treatment of AuNPs, Au/CNTs, and CNTs on HEK-293 cell viability post-48 h treatment. ** *p* < 0.001.

The treatment of AuNPs and Au/CNTs on HEK-293 cells showed a decline in the cancer cell population, but the treatment of CNTs did not show a significant decline in HEK- 293 as in Figure 10. Our results demonstrate that the prepared AuNPs and Au/CNTs showed cytotoxicity against HCT-116 and HeLa cells. Many studies have shown that different nanomaterials and biomaterials produce anti-cancer activities [37,54,55,56]. The cell viability assay based on the in vitro assay showed that the treatment of nanoparticles on normal cells produced significant cytotoxicity, which may be controlled or reduced by using appropriate biomaterials as a combination therapy approach, to reduce the cell toxicity. In future studies, this cell toxicity can be reduced with appropriately functionalized nanomaterials. The impact of different concentrations (2 ug/mL, 10 ug/mL, 20 ug/mL, and 40 ug/mL) of AuNPs, Au/CNTs, and CNTs on cancer cells were examined, it was found that AuNPs, Au/CNTs produced significant cytotoxicity on both HCT-116 and HeLa cells. Whereas different concentrations (2 ug/mL, 10 ug/mL, 20 ug/mL, and 40 ug/mL) of CNTs produced non-significant cytotoxicity on both HCT-116 and HeLa cells. Lower contraction (2 ug/mL) produced less cytotoxicity than higher concentrations (10 ug, 20 ug, and 40 ug) on the cancer cells. 

## 6. Anti-Apoptotic Impact of Nanocomposites

**Figure 11:**
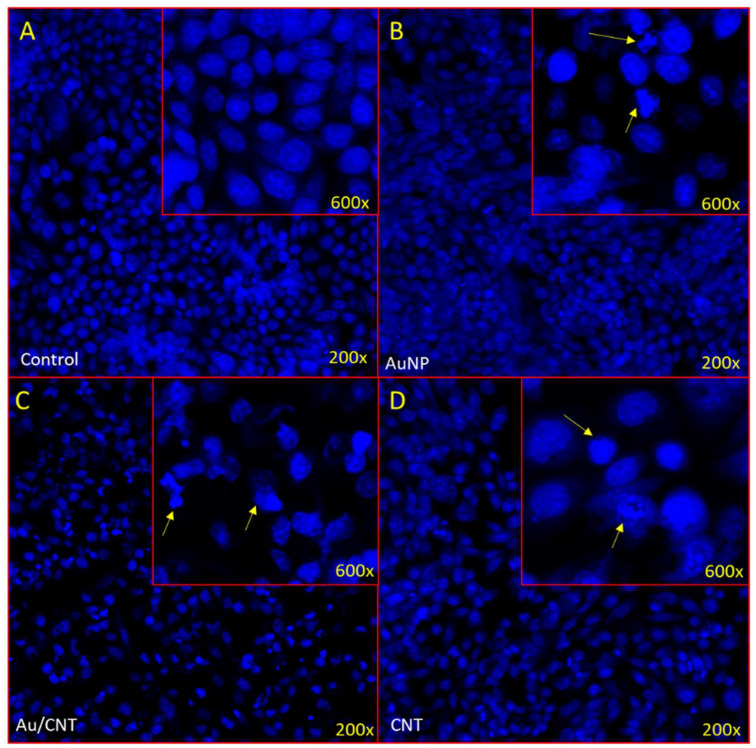
(**A**–**D**): Apoptotic death of colon cancer as revealed by DAPI staining. It shows the impact of AuNPs, Au/CNTs, and CNTs on colon cancer cells (HCT-116). (**A**) shows normal morphology while cells are intact and healthy; (**B**) treatment (40 ug/mL) of AuNP, (**C**) treatment (40 ug/mL) of (Au/CNTs), and (**D**) treatment (40 ug/mL) of (CNTs) showing formation of apoptotic bodies (Arrows). Magnifications 200× and 600×.

Many studies suggest that the treatment of nanoparticles causes significant loss of cancer cells due to programmed cell death [36,37,54,57]. To better understand the reason for cancer cell death, we took HCT-116 cells to examine the cancer cell nuclei by DAPI (4’,6-diamidino-2-phenylindole), which is a blue-fluorescent DNA stain used in identifying apoptosis. The treatment of AuNPs, and Au/CNTs produced a considerably high cancer cell death Figure 11A. In addition, we observed chromatin condensation and formation of apoptotic bodies post-AuNP and Au/CNT treated HCT-166 cells as in Figure 11B,C. The treatment of CNT did not produce any significant change in the morphology of the cancer cell nuclei as in Figure 11D. 

## 7. Conclusions

In this study, nanostructured Au and Au/CNT nanocomposites were fabricated using the PLA technique. The XRD technique confirmed the crystalline nature of the materials. TEM, EDX, and SEM confirmed the morphology of the materials. The AuNPs, Au/CNTs, and CNTs were tested as potential cancer nanotherapeutics on HCT-116 and HeLa using an MTT assay. The result showed a significant reduction in cancer cells post-48 h treatments of AuNPs and Au/CNTs. The treatment of AuNPs and Au/CNTs showed strong inhibitory action on HCT-116 and HeLa cells. However, the treatment of CNTs did not produce a significant decrease in both types of cells, as there was a minor decrease. In addition, the treatment of AuNPs and Au/CNTs also affected the cancer cell nuclei as cancer death occurred because of programmed cell death or apoptosis. The choice of an eco-friendly laser technique and the incorporation of both AuNPs and CNTs as efficient drug-delivery vehicles afforded nanocomposites that possess promising anti-colon and anti-cervical cancer capabilities. 

## Data Availability

The data that support the findings of this study are available from the corresponding author upon reasonable request.
